# Foot involvement in early rheumatoid arthritis: a prospective study of ultrasound features

**DOI:** 10.1186/1757-1146-6-S1-O42

**Published:** 2013-05-31

**Authors:** Lisa Newcombe, Jim Woodburn, Duncan Porter, Sarah Saunders, David McCarey, Monica Gupta, Debbie Turner

**Affiliations:** 1 Institute for Applied Health Research, Glasgow Caledonian University, Glasgow, Scotland, UK; 2 University of Glasgow, Glasgow, Scotland, UK; 3 Department of Rheumatology, Glasgow Royal Infirmary, Glasgow, Scotland, UK; 4 Department of Rheumatology, Gartnavel General Hospital, Glasgow, Scotland, UK

## Background

Foot involvement in early rheumatoid arthritis (RA) is highly prevalent. Our understanding of foot progression and persistence is limited. This study aims to investigate ultrasound features of foot disease in early RA patients over 12 months.

## Methods

Patients with early RA were assessed prospectively for 12 months using high-resolution B-mode and Power Doppler (PD) ultrasound. A cumulative ultrasound score was derived to measure change in the presence of joint effusions, synovitis, erosions, PD, and tenosynovitis between baseline and 12 months. Change in scores was calculated alongside change in global disease (DAS28), disability (HAQ) and foot-related impairment (FIS-RA_IF_) and disability (FIS-RA_AP_) using FIS-RA subscales.

## Results

Thirty early RA patients with a mean ± SD age of 48.8 ± 12.2 years and median (IQR) disease duration of 7.5 (4, 18) months were studied. Over 12 months, patient treatment with disease-modifying and biological drugs increased. Small or stable median (IQR) changes in global disability, foot-related impairment and disability and ultrasound features including joint effusions (-2 (-7, 2)), synovitis (1 (-1, 3)), erosions (0 (-2, 2)), PD (1 (-1, 3) and tenosynovitis (0 (0, 1)) were observed despite a threefold increase in patients entering remission (Baseline: n=5; Exit: n=15). Significant differences (p<0.05) were observed between change in synovitis scores and DAS28 response where synovitis deteriorated in non-responders (3 (0, 5)) and improved with good response (-1 (-2, 1)).

## Conclusions

A trend towards stable and persistent ultrasound features, foot impairment and disability despite an increasing proportion of patients entering remission supports earlier assessment and targeted foot care in early RA.

